# How people diagnosed with borderline personality disorder experience relationships to oneself and to others. A qualitative in-depth study

**DOI:** 10.1080/17482631.2022.2152220

**Published:** 2022-11-30

**Authors:** Christian Moltu, Britt Kverme, Marius Veseth, Eli Natvik

**Affiliations:** a Department of Psychiatry, District General Hospital of Førde, Førde, Norway; b Department of Health and Caring sciences, Western Norway University of Applied Science, Førde, Norway; c Borgestadklinikken, Blue Cross Resource Centre for Substance Dependence Problems, Skien, Norway; d Department of Clinical Psychology, University of Bergen, Bergen, Norway

**Keywords:** First-person, borderline personality disorder, self-harm, emotion, relational, intersubjective

## Abstract

**Background:**

The first-person experiences of people diagnosed with borderline personality disorder (BPD) is an important area of research. It can support clinical and ethical practice, and nuance and expand on insights offered by diagnostic and treatment-oriented research approaches. In this study, we aimed to develop knowledge about how persons who were recently diagnosed with BPD experience being in relationships with themselves and others.

**Methods:**

We conducted in-depth life-world interviews with 12 women recently diagnosed with BPD. The interviews focused on their lived experiences of relationships to self and others. All participants gave their informed consents to participate. We analysed the data with a structured approach to reflexive thematic analysis, conducted as a team-based approach.

**Results:**

We extracted an overarching theme, “Reaching for firm holdings”, that is the most abstract interpretation of participants’ experiences. The five subordinate themes (“Captive of emotions”, “Keeping undeservedness at bay”, “Distrusting oneself”, “Dependence as stability” and “The uncertainty of reaching out”) are specific constituents of the overarching theme, and provide detail and variations across individual accounts.

**Conclusions:**

The results suggest that the experience of relationship to self and others of people recently diagnosed with BPD entails feeling insecure, unsafe and frightened. We report five themes that describe ways participants seek to cope with this situation. The results indicate that their experiences encompass turning to others, or to objects, for feelings of safety. As such, the experience of relationship to self and others in the context of receiving a BPD diagnosis seemed to entail finding and evolving strategies to protect a vulnerable self. Self-harm, suicide attempts and addiction all seemed to be ways of handling and tolerating chaotic and frightful emotions. One major limitation of our study is that only people who identified as female were recruited to participate in the study.

From a professional perspective, borderline personality disorder (BPD) is a severe mental health disorder affecting 0,7–1,8% of the general population (Swartz et al., 1990; Torgersen et al., 2001). The criteria for the diagnosis are a pervasive pattern of instability in interpersonal relationships, self-image and affect and marked impulsivity beginning in early adulthood and presenting in a variety of contexts (American Psychiatric Association, 2013). However, researchers have suggested that there are discrepancies between the objective diagnostic criteria of BPD and how BPD is experienced from a subjective perspective (Flanagan et al., 2010; Kverme et al., 2019; Miller, 1994; Nehls, 1999; Vandyk et al., 2019).

Miller (1994), who interviewed 10 persons about how they experienced living with BPD, found what she described as a chronic feeling of emptiness to be subjectively experienced as an ever present feeling of despair. She further found that the participants did not present themselves as having a disturbed identity. Perseius et al. (2005), who collected diaries and poems and interviewed 10 persons diagnosed with BPD to study how they perceived their suffering, observed ambivalence in the participants’ narratives, as the need for companionship and love conflicted with feeling themselves unlovable. They further described ambivalence between wanting to live and the desire to die. Miller et al. (2021), who explored how people diagnosed with BPD experience chronic feelings of emptiness, described their participants’ disconnection, numbness, purposelessness and unfulfilment as relevant phenomena. Nehls (1999) interviewed 30 persons about the meaning of living with BPD and found that the participants felt misunderstood. Moreover, whereas health workers interpreted incidents of self-harm to be acts of manipulations, participants reported engaging in these behaviours as a means to control emotional pain (Nehls, 1998). These studies suggest that the experience of BPD is complex and varies across situations.

As for studies exploring people with BPD in the context of health care, we can observe both problematic issues and positive development. Historically, Woollaston and Hixenbaugh (2008) and Treloar (2009) reported that health personnel interpreted persons with BPD to be manipulative. Woollaston and Hixenbaugh (2008) interviewed nurses about their experience of treating patients diagnosed with BPD, and found that the nurses used words such as destructive, manipulative and dangerous to describe their experiences with them. Treloar (2009) questioned 140 clinicians about their views of patients diagnosed with BPD, and reported that the clinicians viewed the behaviour of patients with BPD to be manipulative. The study’s participants said the diagnosis of BPD was used as an excuse for bad behaviour (Treloar, 2009). However, since the early 2000s a stronger evidence base suggesting that people with BPD can have good outcome of services might have challenged these perceptions. As one example, Dickens et al. (2019) studied a 1-day educational intervention in collaboration with an expert-by-experience group that reflected recent developments in understanding, treatment potentials and outcome expectations. They report some sustained positive effects of parts of the intervention, but cautioned that more development is needed. McCarrick et al. (2022) interviewed nurses working with people with BPD in an acute ward and found that they indeed experienced frustration and powerlessness, but that they tied this both to the severity of their patients suffering and problems with the organization of services themselves. Ratcliffe and Stenfert Kroese (2021) interviewed service users and managers to explore qualities valued in nursing staff, and reported that investment in the therapeutic relationship, a respectful and approachable manner, and realistic expectations and coping in teams were highly valued. These examples might suggest that although the BPD diagnosis still carries with it problematic stigma, recent developments in knowledge and attitudes are reflected in practice. The results of both of these studies, primarily rooted in the provider perspective, still reflect negative perceptions of persons with BPD, but they offer little information about what living with BPD entails for the person. The problem may still be that lack of knowledge and negative attitudes can contribute to misconceptions about BPD and, in turn, lack of empathy from health professionals.

When the first-person perspective of people with BPD is included in studies, it has often focused on their experiences with a specific treatment setting or with the recovery process (Kverme et al., 2019). For example, Katsakou et al. (2012) interviewed 48 participants in a treatment context, and found that they felt therapy focussed too narrowly on particular areas of functioning, such as self-harm, and that a variety of their needs were neglected. Treatment inflexibility and the need to include a broader set of approaches were also underscored in a qualitative study of clients’ and carer’s perspectives (Barr et al., 2020). Kverme et al. (2019) reported that people in treatment for BPD navigated between developing connectedness and autonomy in trying to make treatment fit their recovery needs. Koivisto et al. (2021), who studied first-person experiences with psychoeducational groups for BPD, reported that information, perspective and being connected to a group were experienced as helpful, whereas experiences with aggression and inflexibility were not. Between studies of treatment experiences, relationality and becoming connected to others seem a pivotal point. However, when an intervention is the focus of a study, the full narrative of the participant diagnosed with BDP can slip into the background. Flanagan et al. (2010) underscored that this is a significant issue: when research focusses on diagnostic concepts or treatment interventions, the subjective experiences of those receiving them tend to be ignored.

This point has been developed by various theoretical approaches. For example, Beverley (2020) used the term hermeneutical injustice to discuss how gaps between collective understanding of a phenomenon and the individual’s experience of living it can limit helpful dialogical practices. Beverley used sexual harassment as an example: before this phenomenon was addressed and established as a construct in common parlance, those who experienced situations that would later be understood as part of this construct had very limited opportunities to talk about their experiences. Kyratsous and Sanati (2017), who specifically addressed the field of personality disorders, used the term epistemic injustice to warn against the consequences of silencing somebody’s capacity as a knower. For people diagnosed with borderline personality disorder, this could, for example, manifest when the dominant discourse (here, for example a diagnostic- or treatment-focus on self-harm or interpersonal dysregulation) silences or has little language for the first-person experiences of horrendous trauma. Natvik and Moltu (2016) discussed how phenomenological studies could alleviate such gaps by allowing empathetic engagement with first-person experiences, and consequently, that such studies might be particularly important in supporting clinical ethics. More broadly, this points to the potential of qualitative research as being reflective, critical, evocative and ethical in the field of mental health (Binder et al., 2016).

Empirical studies emphasize different phenomena depending on whether they build on an intrapsychic or intersubjective epistemology, or, put more simply, whether human phenomena exist within individuals or emerge between individuals. Clearly, the meaning of self-harm will be different between a non-relational and a relational perspective, and nuanced understanding can be lost if empirical findings are not contextualized theoretically. The point of departure for the present study is that human phenomena emerge both within and between, in line with the intersubjective and relational tradition (Benjamin, 1995, 2004; Mitchell, 2000). This perspective has hitherto been more pronounced in clinical rather than empirical work.

Aiming to contribute knowledge that expands on the literature’s suggestion that connectivity and relationships to oneself and to others are core difficult phenomena in borderline personality disorder, this study set out to explore the subjective real-world experiences of people diagnosed with BPD, without focussing on a particular treatment intervention or recovery phase. We, therefore, asked the research question: How do persons who are recently diagnosed with BPD experience the relationships to themselves and to others?

## Materials and methods

### Participants

We established two inclusion criteria. First, eligible participants should have received a BPD diagnosis between six and 18 months prior to the study. Second, to manage recruitment and be in accordance with regulations set by ethical standards, participants had to have contacted a treatment provider, which helped us get in touch with potential participants through invitations to the study. Our exclusion criterion was active psychosis to safeguard the well-being of participants and not be intrusive. We collaborated with four different treatment centres to obtain the sample. Collaborating clinicians at these centres were provided with the invitation letter to distribute to potential participants; the letter explained the scope and focus of the study. Eighteen women were invited to participate in the study, and 12 consented to take part. We did not intend only to include women, but the diagnostic practices for BPD led to women mostly being given this diagnosis. Participants ranged in age from 21 to 37 years. See Table 1 for an overview over participants.

**Table 1.** Participants.

*N*	Sex	Age	Treatment context
7	F	21-37	Active dialectic-behavioural treatment (DBT)
1	F	26	Finalized DBT treatment, prolonged treatment as usual (TAU)
1	F	36	DBT pre-treatment
2	F	22-25	Active Mentalization-based treatment (MBT)
1	F	27	Active TAU inspired by MBT, without the groups

### Researchers

CM is a practicing clinical psychologist and professor of clinical psychology, with extensive experience in recovery research, health service research and participatory research approaches. BK is a practicing clinical psychologist. MV is a researcher and associate professor (PhD) of clinical psychology. EN is an associate professor (PhD) of health science. As a group, the authors share an interest in humanistic and relational health perspectives, participatory research approaches and phenomenological approaches to understanding. None of the researchers had any prior relationship to any of the participants.

### Data collection

We conducted in-depth interviews with the participants, face-to-face, in a separate interview room at the treatment centre with which participants had a contact. Individual in-depth interviews were used to allow for the safe and flexible exploration of participants’ personal experiences (Binder et al., 2012; Knox & Burkard, 2009; Kvale & Brinkmann, 2009). BK conducted all the interviews. Prior to data collection, we developed an interview schedule that aimed for optimal flexibility: we defined some broad questions or themes that we wanted to cover, but focussed on developing prompts, reflections and mirroring under each theme in the actual interview to be able to follow and explore the participants’ narratives and experiences as closely and flexibly as possible. The interview guide is presented in Table 2. Results coming from themes 1 and 4, pertaining to health service and recovery experiences, have been published elsewhere (Kverme et al., 2019). The current paper reports analyses following theme 2 and 3. The twelve interviews lasted 45–150 minutes, and all were audio-recorded and transcribed verbatim for structured qualitative analysis.

**Table 2.** Interview schedule.

Theme	Interview questions
**Introduction to interviews**: Thank you for agreeing to participate in this study. We wish do explore and understand how it is to receive a BPD diagnosis, and how it is to live with it. We are very interested in what you have to tell about your experiences, and I have a set of questions that I would like to ask, that might help us understand how this has been for you. I hope that we can talk freely, and that under each question we might explore your experiences.	
**Theme 1**. The personal narrative about receiving a BPD diagnosis	Can you remember the day that you got the BPD diagnosis? Can you please tell me how you experienced it? Who told you? How did they tell you? Was it something with that situation that you remember particularly well? What were your thoughts in that situation? How did you feel?
**Theme 2**. The relational and emotional meaning of receiving a BPD diagnosis	How are you talking to others about having received a BPD diagnosis? How do you experience their reactions? How do you experience sharing having received a BPD diagnosis with others? Who do you feel you can discuss such matters with?
**Theme 3**. The personal experience of living with a BPD diagnosis	What has it meant to you to receive this diagnosis? How do you experience your relationship to other people? Can you please tell me in depth how you experience the issues that you understand as connected to the diagnosis in your everyday life? What is most difficult for you? What are your strengths?
**Theme 4**. Experiences with health services	From your own experience, what would be good advice to people who are to give someone a BPD diagnosis, to make this as good an experience as possible for the one who receives it? From your own experience, what would be good advice to people who receive a BPD diagnosis on how they can care for themselves and use this situation in a good way for themselves?

### Data analysis

We used a team-based structured approach to rigorously analyse the data material (Binder et al., 2012), which was influenced by phenomenological epistemology. To structure the steps and presentation of the data analysis, we employed a framework of thematic analysis (Braun & Clarke, 2006). This approach aims to establish themes across individual accounts that detail and discuss meanings, convergences and divergences within narratives. We proceeded through these concrete steps: First, all the authors read the full data material closely to get an overview of the content and variations within it, and noted preliminary ideas about important points. Second, CM, BK and EN met for an analytic seminar to discuss and compare first stage notes, look for immediate patterns and establish a joint focus for structured analyses. Third, BK implemented the focus established in step two for a detailed coding of the data material for preliminary themes. At this step, codes where at the meaning unit level, for example the code “Not trusting experiences” tied to the utterance “If I have been out and had a good night, I don’t know what to make of it, I don’t know where to place it, was it something wrong? I just can’t seem to get it in place. The same with relationships”, and the code “depended on structure” tied to the utterance “If I don’t exercise by eight o´clock, and if, by some reason, the time has gone to half past nine, then my day is ruined!” Fourth, CM, BK and EN met for a full-day analytic seminar to establish the preliminary themes emerging through the coding in step three. In this stage, codes were grouped by similarity to establish preliminary themes. Adjustments and additions were implemented by consensus. Fifth, BK refined the analysis based on the seminar’s consensus and checked all the adjustments fit with the data material. Sixth, MV, who had not been part of the coding and analysis team, thus far, audited the resulting thematic structure and its correspondence to raw data independently, performing the function of a critical auditor (Hill, 2012).

### Ethics

The project was submitted for full evaluation by the Regional Ethics Committee for Medical Health Research (REK). REK approved the project (reference number 2015/882). During the study, we were particularly mindful that we were inviting participants with a potentially high degree of suffering and a range of traumatic experiences. We, therefore, aimed to safeguard their well-being by requiring that they had access to an ongoing treatment relationship as part of the inclusion criteria, and by providing a debriefing with a trained psychotherapist during and after interviews if they wanted it. None of the participants expressed adverse experiences from the interviews or a need for follow-up or debriefing.

## Results

**Figure 1.**
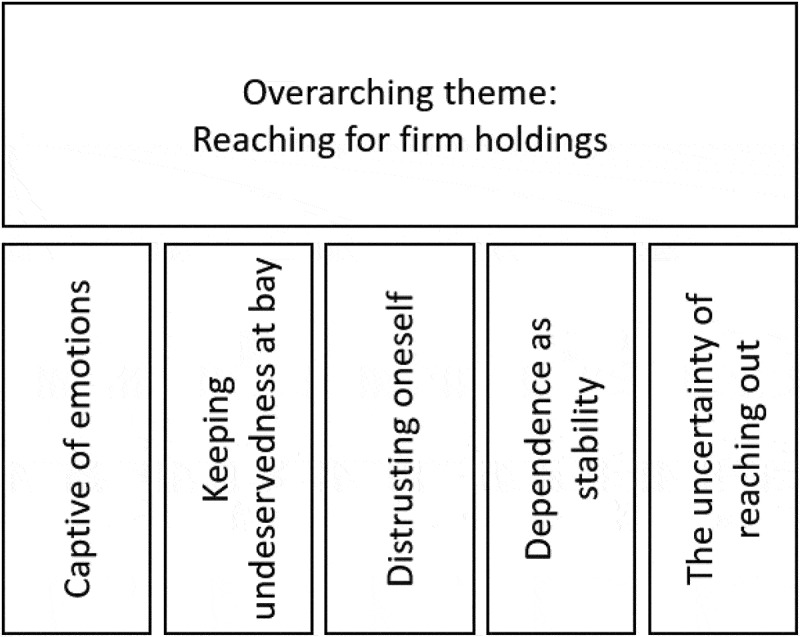
Visual overview of results.

An overarching theme of “Reaching for firm holdings” was at the centre of the experience of relationships when living with BPD. This theme highlights the meanings in the subordinate themes: “Captive of emotions”, “Keeping undeservedness at bay”, “Distrusting oneself”, “Dependence as stability”, and “The uncertainty of reaching out”. Figure 1 provides a visual overview over the findings. These subordinate themes represent variations of the overarching theme. There was not one way of experiencing the relationships to oneself and others in the context of a recent BPD diagnosis, but patterns of experiences converged on the shared overarching theme, with more or less of the subordinate themes expressed across the individual accounts. When detailing the themes in the following sections, we try to stay as close as possible to the language used by participants, in order to have the presentation grounded in their experiences.

### Reaching for firm holdings

Seeking stability and security meant actively trying to care for oneself in a situation of profound instability and insecurity, both in the relationship to self and in relationships to others. However, “reaching for” also implied a sense of anxiety, as the participants constantly feared losing that which they had taken hold of. The participants seemed to experience themselves as beings of confusion and chaos. They described feeling fragmented, not fully knowing who they were, and expressed insecurity about their own existence. Importantly, not knowing who they were in themselves also involved not knowing who they were for others. The participants described fearing their own thoughts and emotions, as they experienced them as uncontrollable and unbearable. Reaching for someone or something that could hold the participant seemed to create a temporary distance from these intense experiences, giving the participants momentary relief. Reaching for could entail taking drugs, consuming alcohol, self-harm, obsessing about being perfect or reaching for confirmation from other humans, but it also involved seeking connection with and trying to trust other persons. Variations in how participants described reaching for firm holdings and what this experience entailed for them is explored in more depth in the constituent subthemes.

### Captive of emotions

Participants described their emotions as an intertwining of different voices shouting over each other and creating an unbearable chaos of thoughts and agony within their bodies. Experiencing emotions were mostly described as a relationship to oneself. Emotions were intense, sweeping over the participants like a wave, and they saw no escape from this surge. These experiences left the participants desperate for calm, and they needed to escape from their captivity. Emotions were not necessarily distressing, as they could also be experienced as elevating and uplifting, including the experience of joy. The issue was their uncontrollable and unpredictable nature, and how they sometimes felt like an autonomous part of the self. The following quote illustrates these experiences; which were seemingly coming “out of the blue”, with no trace of what led to them. In quotes, we use (…) to signify that redundant text has been omitted for clarity,

I can wake up feeling depressed, and then three days goes by, and I´m high up. I have no middle, either I´m down or I´m up. (…) Either I´m happy and blue eyed and the world is really nice and everything is just ‘YES’, or it´s death, destruction, war, kill me! I´m like that.

This participant described being at the mercy of her emotional states as highs and lows would come and go, leaving her longing for the arrival of a balanced mood in-between them. The participants had little control over, or understanding of their own emotional reactions. It could feel like a relationship to a part of self with immense influence, but that was very hard to predict. Emotional distress was experienced as something taking control over their body and mind, not as nuanced experiences that could be divided into meaningful entities, as illustrated in the quote below.

I did not have any words to describe my feelings, my head and body did not connect, I felt separated, my head was just chaos, I did not know how to put things into words, I didn´t know, I knew anger though, if I felt angry.

Participants experienced emotions as a peripheral or estranged part of self, taking control. This lead them to feel powerless in their subjective, or recipient, position. The disempowered experience is underscored by the fact that many participants expressed that by inflicting pain on themselves, they regained a position of being able to cause emotions, not only receive them out of the blue. This could give them a momentary feeling of control in relation to themselves and meant that they could challenge the agony within, forcing it to release its hold on them. Self-harm seemed, as such, to be an instrument that enabled the participants to regain authority in the relation to their emotional experiences. In a similar vein, one participant described that she used food as a way of taking control over her emotions, using her body as a vehicle for both reducing pain and causing it, as it would make her gain excessive weight, leading her to feel embarrassed about her appearance. Furthermore, the possibility of taking one´s own life was also seen by participants to be about regaining control, as it could offer emotional relief and calmness. For example, by hitting her head against the wall, the participant would lift herself out of the emotional pain she was in. The quote below illustrates why.

The pain I feel inside can be so intolerable to bear that my initial reaction is to harm myself.

One participant described in the quote below that she experienced the option to end her life as soothing, as if the thought of it was soothing in itself.

I´m thinking that when it gets too scary, there are two options for me. One is to get really ill with my eating disorder again, that life just, you almost die, or I could kill myself. If life gets too scary then those two options are something I know that I have, almost comforting.

Self-harm and thoughts of suicide seemed to be experienced as an exit sign showing the participants an actionable way out of their emotional captivity and helping them to take control over an emotional situation they perceived as uncontrollable.

### Keeping undeservedness at bay

Many participants described a need to be perfect, without having a clear sense of what ideal they were aiming to reach, or what being perfect would entail. It was almost as if they were wandering in blindness in their effort to be flawless. They saw the need to be perfect to stem from a feeling of being flawed, of being damaged and being worthless. Striving for perfection seemed to be about keeping these feelings at arms length. The sense of a worthless self structured relationships with other people.

For example, one participant looked to the people around her to help her create a distance to her sense of worthlessness, needing them to confirm that she was good enough, as illustrated in the quote below.

I would feel insecure a lot; I still do. I need confirmation several times a day, to confirm that I do things right, because I will judge myself a lot. I have this perfectionist in me; if it´s not perfect then it´s not good.

The need to be perfect seemingly had a destructive hold on the participants, as they said that feelings of being flawed and worthless would wash over them again as soon as they did not live up to their high demands, or they did not feel validated by others. Some of the participants described that they would punish themselves for their own shortcomings, torturing themselves by leaving marks on their body, or speaking hurtful words to themselves about how they did not deserve to be alive. The participants described their inner dialog as disapproving and constantly demeaning. As such, this theme relates to the theme “Captive by emotions”, but is also more specific, in that the intersubjective relationship to others is a more prominent constituent in it. Self-worth seemed to be self-worth influenced by the perception of others. Consequently, rebelling against it seemingly caused anxiety instead of, or in addition to, a sense of control, as illustrated by the following quote. One participant described experiencing guilt and shame after a blissful moment when she dared to challenge the voice that told her she was worthless.

(.) When I am really high up, I can feel invincible. No one can defeat me; what I did was fantastic, you know. But, when I come down from that high this thought is suddenly there: “How could you think that way about yourself!” Then, I feel the urge to punish myself for being positive.

Another participant related a more stable sense of undeservedness.

(.) I don´t deserve anything good in life; I deserve the mud on the bottom of the sea. I am the mud, I am mud! That´s how I have felt about myself for years! I don´t deserve any joy, I don’t deserve anything good! I deserve all the bad things happening to me.

This participant seemed to have resigned in her fight against feeling damaged, instead, welcoming it with open arms, making her feelings of self-loathing something to worship, and sacrificing her own chance of bliss to serve it. Her aim was not to create a distance from her feelings; she wanted to confirm and give nourishment to them, making them draw even closer. This experience seemed to underscore the intolerable tension of intersubjectivity by illustrating how the participant had needed to surrender to relating to herself as a non-changing object. When relating to herself as only unworthy, rather than a self with hope and relational aspirations, the participant seemed protected from the unpredictability that comes from relating to others as subjects.

### Distrusting oneself

The participants described an experience of not being anchored, as if they were floating around within themselves and among others. They described an experience of being unable to hold, to comfort or to validate themselves. To utter words like “you are good enough” seemed impossible as they themselves could not be trusted. In such processes, it seemed confusing for the participants who the speaker- and who the recipient self is, and who could be trusted. The participants described constantly doubting their own inner world, their thoughts and emotions, as well as their perception of the world around them. This distrust made a participant question every social situation she was in, for example having lunch with friends or going out. She expressed having difficulty placing these experiences in her relationship to herself and her relationships to others, not being able to make sense of, or understand their meaning.

If I have been out and had a good night, I don’t know what to make of it, I don’t know where to place it. Was it something wrong? I just can’t seem to get it in place. The same with relationships, some might think it´s weird, but I have never had a boyfriend! I have been ill since I was a teenager, so regular things like eating lunch with someone can be difficult. It´s difficult to explain, but I have difficulties sorting; it´s almost easier when things are difficult.

One participant experienced herself being aggressive with her family, careless with her friends and depressed when alone. Integrating these different sides of her person into one seemed impossible. She experienced feeling torn, finding her self to be unclear and distant. The experience of being illusive to oneself could be intensely felt. One participant experienced herself vanishing when alone, losing grip on her own being and feeling herself slipping through her fingers.

I struggle a lot with the fact that I lose myself when I am alone. Life can become hopeless and I can’t seem to do anything, even if I would like to draw or something, I rarely manage to do anything when I´m home (…) It’s just about passing the time until it´s time to go to bed.

Not trusting one´s own being made the participants reach out to fellow humans to make the world around them safer and less frightening, as illustrated in the quote below. This participant described an experience of not being anchored, of falling without reaching the ground. This made her desperately grip on to anyone to have them watch over her, holding on to them so tight that she would feel herself anchored down.

It might be my diagnosis, that I need to be watched over, I can feel that I´m free falling, there is nothing to hold, there is no anchor.

Some of the participants described having difficulty even placing themselves, to find footing within themselves, to know who they were as persons, and to experience themselves the same over time. The participants described a feeling of pretending to be someone, ever changing to what seemed to be expected from the outside world.

### Dependence as stability

Many of the participants told us that they engaged in dependent relationships both to things and to other people. It could be alcohol, drugs, love, exercise or relationships. Dependency seemed to create a place where personal and relational difficulties could fade into the background. However, dependence could also be limiting in setting boundaries for how the participants unfolded themselves in their lives. The quote below illustrates this ambiguity; this participant exercised every day at the same time; when she ran or went hiking her thoughts would wander away from her problems and her anxiety seemed to subside. However, she would be overwhelmed by guilt if she missed her workout by one hour, spiralling her into destructive thinking about her life and how she experienced herself as a being burden to society.

If I don’t exercise by eight o´clock, and if, for some reason, the time has gone to half-past nine, then my day is ruined! Then everything goes to hell; then I´m stressing. It takes as little as that, one hour out of schedule, and my day has gone to hell! (...) I always need to have something to occupy me. If I just sit down on the couch, I realize how little control I have over my life, all the things that aren’t as they should be. I feel really ashamed when I´m not working; I feel like a parasite.

Addiction became something dependable in the midst of confusing relationships to oneself and to others. Like a trusted friend, addiction was there stretching out a hand, stable, known and predictable. It was something to look forward to, filling the days. It could substitute needs; it could take loneliness away for a period of time and give comfort. At the same time, dependence was an enemy hiding in its disguise of being a trusted friend. A participant described easily turning to prescription pills, which gave her momentary relief in her daily struggles.

I have a really addictive personality; I easily get dependent on people. I can become really obsessive and think that, “If I only have her as a friend, others don’t matter”. I have been in love with people I don’t even know and responded to that by getting high, drinking alcohol or taking pills. I have been addicted to anxiety pills; I have used them to not feel the intense feelings inside me, or to handle certain situations better. So, I easily get addicted.

Also in this theme, self-inflicted pain was experienced as something that gave emotional relief, and it was also described as having a drug-like quality, as it rewarded the participants with a euphoric feeling. A participant described that harming herself became all-consuming and challenging to let go of, as it held the excitement of taking her to the utter edge of life, balancing her between life and death. In the quote below, she vividly described one of her self-harming episodes and her longing to experience it again.

Just before I lose consciousness, what I remember in that moment is feeling peace and a bright light; then, I close my eyes and it´s quiet. God, I would want to be in that light, that peace in that moment, that second. I can understand drug addicts and alcoholics, their urge for their own self-harming if that’s how it can be described.

### The uncertainty of reaching out

Other people were experienced as ever changing and insecure. However, the participants would reach out in relationships to others for stability and security, to balance their hard-to-hold and insecure relationship to self. The relationship to others and the relationship to self seemed deeply intertwined. The participants’ needed to reach outside themselves for security, which left them in fear of losing their hold. This fear seemed to lead some of the participants to push their friends and families to the limit, testing their love and tolerance, testing their security. In the illustrative quote below, a participant described feeling so unsound in her own being that she would be overwhelmed by a need to be nurtured by someone secure and dependable.

It might be what this personality disorder entails, because all I wanted was that someone would take me home with them, you know (cries). Take me home and take care of me. That’s what I wanted and still want, but that’s not good, or what I want does not go together with what is actually good for me.

In her effort to extinguish the loneliness burning within, this participant clung on to others. In this dynamic, the other person’s autonomy seemed scary. She experienced that she was suffocating them, unwittingly pushing away those she desperately needed to be near. Paradoxically, her strive for belonging and closeness seemed to be the same thing that hindered her from obtaining them.

My challenge is that I become so wrapped up in other people, I´m very lonely and can grab on to people, you know, I´m very lonely, I feel very lonely. I just want to be with other people, so I become too much, so then it´s a big thing when people reject me you know.

Interpreting relationships to others could be very confusing. One participant described trying to control this uncertainty by staying one step ahead of it. The quote below illustrates how she monitored her friends, searching for signs in their facial expressions to interpret their emotional state. She chased clues to how to behave and understand the social situation she found herself in. She described being overwhelmed by insecurities when the mood of the situation would shift, fearing that she did something wrong to cause the sudden change.

I have thought about how much I look at the body language of those I talk to while I´m talking to them. If I can see that he or she looks angry, or I get those thoughts, like “Did I say something wrong?” Or if I say something and that other person makes a sound and looks upset, then I´m like “Did I say something wrong?”

Even though reaching out held uncertainties, relationships to others was the strategy that participants said they used to tolerate and bear that which they experienced as being intolerable inside them. At times, they felt locked in a painful paradox where it felt intolerable to be with others, but also to be without others. In spite of this pain, most participants described that they kept trying, as they experienced that reaching out helped them to hold on to their lives until the next day.

## Discussion

Summarizing the presented themes and their descriptive content, our results suggest that the experience of relationships to oneself and others for people recently diagnosed with BPD entails feeling insecure, unsafe and frightened, and being unable to find shelter from these experiences within oneself. The results further indicate that their experiences encompass turning to others or to objects for feelings of safety. As such, the experience of intersubjective relationships in the context of receiving a BPD diagnosis seems to entail finding and evolving strategies to protect a vulnerable self. Self-harm, suicide attempts and addiction all seem to be ways of handling and tolerating chaotic and frightful emotions. Self-harm seems to be complex, as it was used to take control over emotions in a self-self relationship as well as for discipline and regulating self-worth in self-other relationships. The latter experiential domain entails feeling undeserving and shameful at the core of one’s personhood. Paradoxically, these feelings also seem to be motivating, pushing the participants to develop towards achieving what they set their mind to.

Considering our results through the lens of relational- and intersubjective psychological theory might help underscore the nuance achieved with the focus on relationships to oneself and others in this paper. Prior research suggests that BPD is associated with insecure or dysregulated primary attachment relationships (Fonagy & Allison, 2014; Fonagy et al., 2004), suggesting an association to relational trauma in formative years. In relational theory, intersubjective development and safety and tolerance in both one’s own and the other’s subjectivity develops mutually and reciprocally by processes of marked mirroring (Bateman & Fonagy, 2004; Benjamin, 2004). Subjective safety and tolerance for emotional experiences emerge in a safe relationship to another human being who recognizes, gives meaning to and tolerates them. In lieu of such a relationship, or if this safety is shattered by trauma, alternative strategies are needed to protect the self. Relational approaches (Aron, 1996; Benjamin, 1995) have shown how such experiences lead to relationality where both self and others are experienced as objects. Objects can either be controlled, or will exert control. In a seminal essay, Benjamin (2004) describes this relational world as “doing, or being done to”, a process in which both the sense of self, and the sense of the other’s self, are obscured. When we explore the phenomenon of relationships to oneself and others for people who have been diagnosed with BPD, this theoretical perspective seems helpful. For example, in the theme “Captive by emotion” central experiential processes seem to be about being controlled by, or controlling, emotions, rather than experiencing them as an integrated part of self. In the theme “the uncertainty of reaching out” similar dynamics emerge. The needs related to others seem static or objectified, in the service of stability, albeit unsustainable over time. Relating to the other as a subject, and by that one who can leave, disappoint and have needs, seems to be horrific in the participants’ experience. Phenomena discussed in this study’s results converge around relating to one’s own and the others’ subjectivity. Exploring experiences through the lens of relationality adds nuance to empirical studies, reporting for example that people diagnosed with BPD feel misunderstood and that health care workers talk about them as manipulative, by contextualizing such end-points within an intersubjective perspective.

The findings suggest that the experience of relationships in the context of having received a BPD diagnosis is both qualitatively different from and similar to what has been described in studies of the experiences of people who have been given other diagnostic tags. For example, Sørensen et al. (2019) studied the experiences of persons with avoidant personality disorder, and reported that participants longed for connection, but dreaded getting close and often chose “being alone, for better or for worse”. While alienation from others and loss seem to echo through both studies, the emotional intensity and the relational actions individuals take to move through this dilemma seem to be very different. Our results suggest that persons with BPD develop strategies to protect themselves and help them tolerate a state of inner chaos, but they find it difficult to resort to isolation. In our study, relationship to others seem to both be entwined in the intersubjective suffering, and also the experienced way forward. Sørensen’s et al. (2019) study also found that relational alienation and insecurity was followed by, or followed, a struggle to achieve a sense of oneself as a person. Outside the focus on personality disorders, Hjeltnes et al. (2015) studied the lived experiences of 29 people diagnosed with social anxiety disorder, and described how withdrawal, loneliness and hiding from others were core experiences across individuals. Their participants, while similar in their experiences of relational isolation, had a stronger hope of potentially getting out of it, given the right circumstances, as they found greater soothing in their own relationship to themselves. Moreover, they reported being more stable in a silent and gloomy suffering, rather than experiencing rapid shifts and emotional extremes.

On the more specific side, our results describing that self-harm is experienced as a strategy to protect oneself and tolerate emotional experiences, find support in results reported by Perseius et al. (2005). They found that episodes of self-harm tended to occur at times of intense emotional pain, and concluded that self-harm could be seen as a means to ease emotional pain and make it tolerable. Nehls (1998) findings support this conclusion as their participants explained their episodes of self-harm as a way to control emotional pain. These earlier studies are supported by more recent work, underscoring the affect regulatory, anti-dissociative functions of self-harm alongside communicative- and self-punishment functions (Reichl & Kaess, 2021). A recent systematic review suggests that self-harm is a regulatory strategy for severe experiences of emptiness (Miller et al., 2020). One very important nuance here is that an explanation of self-harming behaviour as regulation emotions and pain gives a different perspective than what is a reified diagnostic criteria, where fear of abandonment is held forth as a primary motif for self-harm (World Health Organization, 2018). This rather minor difference seems important: the latter more readily lends itself to interpret self-harm as relationally manipulative (you only do this, or threaten to do this, to make me stay with you, or to lock me in one particular relational position with you, rather than allowing me relational freedom), whereas the subjectively based understanding is easier to empathize with (you are in so much pain right now). As testimony to this being important, research has indicated that health personnel say patients with BPD are manipulative (Treloar, 2009; Woollaston & Hixenbaugh, 2008), suggesting that diagnostic formulations influence interpersonal interpretation. Our results potentially add a helpful perspective to these processes.

Our results suggest that the experience of life in the context of a BPD diagnosis includes the experience of being lost, in an intimate pre-reflective sense, in the relationship to one’s own self. This feature is described as identity disturbance in diagnostic manuals (World Health Organization, 2018). However, participants in a study such as this one give added nuance to the picture of this disturbance. In particular, participants experienced themselves as illusive and difficult to hold and undeserving as a self. They would momentarily find themselves in relation to another person or thing, become scared about becoming dependent on that person or thing, only to lose themselves again later. On a similar theme, Miller (1994) found that feeling inadequate in relationships to others was central to the experience of BPD and suggested this to mean that the DSM criteria of identity disturbance entailed an experience of oneself as impaired. Furthermore, the experience of being elusive to oneself could also be connected to participants describing themselves as free falling and disappearing. The point we are trying to make here is simple, and echoes what was illustrated by Natvik and Moltu (2016) for the phenomenon of isolation for people with social anxiety, using a study by Hjeltnes et al. (2015) as a case. Diagnostic discourse and practice, while helpful for a variety of purposes, might contribute to the loss of nuance in understanding people’s lived experiences. Too easily, what is reified as core components will guide the interpretation of a person, with the risk of contributing to epistemic injustice (Kyratsous & Sanati, 2017). First-person research contributes additional information by describing variation and depth in experiences, which might enhance potential helpers’ empathic imagination in meeting with people who suffer, and provide those who suffer with a wider linguistic horizon to express their experiences.

### Limitations

The study only included participants identifying as female. This study, therefore, fails to explore how participants identifying as male experience living with a recent BPD diagnosis. Moreover, our recruitment strategy allowed us to invite people in a help-seeking phase who had relatively recently been given a diagnosis. This suggests that, as a group, our participants would have a relatively high degree of suffering and who were in some form of active relationship vis-à-vis their own problems. Readers should be mindful of this limitation, and be careful making generalizations from these results to the larger group of people who have received a BPD diagnosis. Some of them might have a long-standing experience with both involving themselves in treatment and working towards stability, and have very different experiences than what was shared by our participants. Some of the participants found themselves recovering from their problems when the interviews were conducted. Therefore, the findings, to some extent represent their experiences living with BPD told in retrospect. Lastly, even though we seek, as researchers, to meet the data material without preconceptions, three of us are influenced by our knowledge as psychologists. We have worked, individually and as a group, on reflexive practices (Alvesson & Sköldberg, 2000; Binder et al., 2016; Maso, 2003) to remain open towards the participants’ experiences. Moreover, one researcher in our group has a different professional background, allowing her to contribute to increased transparency by probing verbalization of implicit assumptions of what living with BPD entails. Finally, one member of the research team did not do clinical work, thus, allowing him a freer perspective in discussions.

## Conclusion

We have studied the experiences of relationships to oneself and others in 12 women who were recently diagnosed with BDP. We report five themes that describe ways participants seek to cope with this situation: “Captive of emotions”, “Keeping undeservedness at bay”, “Distrusting oneself”, “Dependence as stability”, and “The uncertainty of reaching out”. The experience of relationships to oneself and others in the context of receiving a BPD diagnosis seemed to entail finding and evolving relational strategies to protect a vulnerable self. Self-harm, suicide attempts and addiction seemed to be ways of handling and tolerating chaotic and frightful emotions.
